# Therapeutic effect of Shinkiwhan, herbal medicine, regulates OPG/RANKL/RANK system on ovariectomy-induced bone loss rat

**DOI:** 10.20463/pan.2020.0017

**Published:** 2020-09-30

**Authors:** Il-bok Seo, Kang Pa Lee, Sun-young Park, Sang-hyun Ahn

**Affiliations:** 1 Department of Anatomy, College of Korean Medicine, Semyung University, Jecheon, Republic of Korea; 2 Research and Development Center, UMUST R&D Corporation, Seoul, Republic of Korea; 3 Department of Physiology, College of Korean Medicine, Semyung University, Jecheon, Republic of Korea

**Keywords:** osteoporosis, osteoprotegerin, p-JNK, receptor-activator of nuclear factor kappa B ligand, Shinkiwhan

## Abstract

**[Purpose]:**

Although physical activity is required to prevent or ameliorate osteoporosis, medicine prescription should precede it, since it may be limited in severe osteoporosis patients. Furthermore, osteoporosis has a great effect on physical activity disorders that accompany fractures and pain, and therefore, research on treatment or prevention to decrease the number of patients is required. The purpose of this study was to discover candidate substances from natural products with an effective pharmacological action and to prepare basic data to help patients.

**[Methods]:**

To prepare the osteoporosis model, ovariectomy (OVX) was performed using surgical methods. The prepared prescription [Shinkiwhan (SKH), a Korean medicine] was administered orally at a dose of 210 mg/kg/day for 8 weeks. After completion of the animal experiment, the bone mineral density (BMD) was analyzed using double-energy X-ray absorptiometry. The analysis of the effect of drugs on bones was performed using histological analysis and immunostaining.

**[Results]:**

SKH increased the BMD in the OVX rats. Furthermore, SKH significantly increased the expression of osteoprotegerin and downregulated receptor activator of nuclear factor kappa B ligand and phosphorylation of c-jun N-terminal kinases in the bones of the OVX model.

**[Conclusion]:**

Our findings suggest a protective effect of SKH against BMD loss in the OVX model.

## INTRODUCTION

Physical activity is effective in preventing and treating diverse diseases. However, westernized diets, sedentary lifestyles, and an irregular physical activity are increasing the incidence of disease in modern people. In particular, older people and postmenopausal women have an increased incidence of osteoporosis due to their inactive lifestyle. Osteoporosis is a representative disease in which bone dysfunction is characterized by a bone mass decrease and an abnormal bone microstructure [1]. According to the National Health & Nutritional Examination Survey, the prevalence of osteoporosis in the United States is estimated to be about 5 million people [2,3]. In particular, the prevalence rate among women who are over 50 years of age is rapidly increasing worldwide [4]. Therefore, it is urgent to diagnose osteoporosis, recognize the need for prevention and treatment, and find a treatment solution.

The skeletal system contains bones, ligaments, and cartilages that play multiple roles in the body, including the protection of internal organs and body structures and enabling movement [5]. Bones support the metabolic incorporation that serves to safely store essential minerals and blood cells [6]. Bones maintain their function through bone remodeling by continuously performing the roles of destroying and forming the bone, done by osteoclasts and osteoblasts, respectively [7]. Osteoprotegerin/receptor activator of nuclear factor kappa B/RANK ligand (OPG/RANK/RANKL) system is tightly involved in bone remodeling. RANK expresses bone activity, and RANKL promotes osteoclast differentiation [8,9]. Additionally, OPG plays a role in inhibiting osteoclast differentiation in the RANKL signaling system [10]. Hence, the analysis of changes in the OPG/RANK/RANK system also provides important clues for discovering osteoporosis treatment candidates.

The most useful method of osteoporosis diagnosis is by measuring the density of the lumbar spine and femur using double-energy X-ray absorptiometry (DXA) [11]. After diagnosis, the therapeutic option is to increase bone mineral density and decrease the frequency of fractures, if sufficient calcium is consumed. In addition, vitamin D intake is not only necessary for calcium absorption in the intestine, but also plays an important role in maintaining bone and muscle function and body balance. In pharmacological therapy, drugs that act as estrogen agonists or antagonists (raloxifene and bazedoxifene) and powerful bone resorption inhibitors (alendronate, risedronate, ibandronate, and zoledronate) are approved and used [12-17].

Natural products, such as plants, animals, microorganisms, and those of metabolic products may contain compounds that exert pharmacological activities that inhibit bone disease [18]. In Korea, there are many candidate substances that can make bones healthy in traditional oriental medicine. Osteoporosis requires a long-term treatment, and we believe that research to find natural products that help in osteoporosis treatment is necessary. However, oriental medical research has not yet been fully established. In the present study, therefore, we investigated the anti-osteoporosis effect of the natural product Shinkiwhan on osteoporosis in an ovariectomized mouse model. Our findings provide basic information for osteoporosis treatment.

## METHODS

### Extract of Shinkiwhan (SKH)

Shinkiwhan, a Korean medicine, was obtained from Jungwoo medicines (Chungcheonnam-do, Korea). *Rehmannia glutinosa* Liboschitz ex Steudel (15 g), *Dioscorea batatas* Decaisne (7.5 g), *Cornus officinalis* Siebold et Zuccarini (7.5 g), *Schisandra chinensis* (Turcz.) Baillon (7.5 g), *Alisma orientale* Juzepzuk (6 g), *Paeonia suffruticosa* Andrews (6 g), and *Poria cocos* Wolf (6 g) were boiled in 2,000 mL of distilled water at 100°C for 3 h, and then, filtered. The decoction was reduced to 50 mL using a rotary evaporator. To obtain an extract of SKH, the supernatant was lyophilized at −60°C.

### Animal care and animal study

Experimental animals were 7-week-old female Sprague-Dawley rats (Sam Taco, Gyeonggi-do, Korea), which were used for experiments after acclimatization for 1 week. All experiments and animal care were performed in accordance with institutional guidelines (SEMCARE 16-06-01). Rats were divided into three groups (normal control: CON; negative control: OVXT; treatment group; SKHT). The CON was sutured without ovarian resection after laparotomy. The osteoporosis animal model was prepared using a surgical method. After resection of the rat ovaries, OVXT and SKHT were administered normal saline or a sample solution (a dose of 210 mg/kg/day) for 8 weeks. Experimental animals were kept in a standard cage in a breeding room maintained at a constant temperature of 25 ± 2°C, a humidity of 55 ± 5%, and 12- hour light/dark cycles, with standard feed containing 1.2% calcium and 0.8% phosphorus (DAMOOLSCIENCE, Daejeon, Korea).

### Analysis of bone mass

After 8 weeks of OVX, anesthesia was performed using diethyl ether, and the femur was removed. The muscles attached to the femur were clearly removed and fixed in 10% neutral buffered formalin at room temperature for 24 h, followed by analysis. Bone density was analyzed using DXA (InAlyzer, Medikors, Seoul, Korea).

### Safranin-O staining

The bones were decalcified using a decalcification solution (Sigma, ST, MO, USA) for 12 h. The bones were washed and embedded in paraffin. The embedded bone samples were sectioned in 5 μm-thick slices. The sections were washed using a xylene solution and dehydrated using serial concentrations of ethanol (100%, 90%, 80%, 70%, and 60%). To observe the bone matrix, the sections were stained with 0.1% Safranin O solution for 5 min. Images were captured using an inverted microscope (Nikon, Tokyo, Japan).

### Immunohistochemistry

Immunohistochemistry was performed as previously described [19]. To detect the expression of specific proteins, such as the phosphorylation of JNK, RANKL, and OPG, the sections were stained with specific antibodies. The 5 μm-thick sections were treated with proteinase K (20 μg/mL) for 5 min and incubated with 10% normal goat serum for 4 h. The sections were incubated with antibodies, such as anti-pJNK, anti-RANKL, and anti-OPG (Santacurz, TX, USA) at 4°C overnight. The sections were then incubated with the biotinylated secondary antibodies. Images were captured using an inverted microscope (Nikon, Tokyo, Japan). The photographs were analyzed using ImageJ software.

### Analysis of gas chromatography and Mass spectrometry

GC/MS analysis was performed as previously described [20], using an Agilent 6890N GC/5975i MS instrument (Palo Alto, CA, USA) and a DB5-MS capillary column (30 m × 250 μm, 0.25 μm film thickness). Helium was used as the carrier gas at a flow rate of 1 mL/min. The injector port and interface temperatures were 280°C and 300°C, respectively. The gas chromatography oven was maintained at 40°C for 2 min, increased to 230°C at a rate of 5°C/min, and then kept constant at 300°C for 5 min. The split ratio was 1:10, and the mass range used was 40 - 800 m/z.

### Statistical analyses

Results are expressed as mean ± standard deviation (n = 6). Multiple comparisons were performed using oneway analysis of variance, followed by Tukey’s *post-hoc* test (GraphPad Prism ver. 4.00 for Windows, GraphPad, CA, USA). *p*-values < 0.05 were considered statistically significant.

## RESULTS

### SKH inhibits the loss of bone mass in OVX rat model

**Figure 1.**
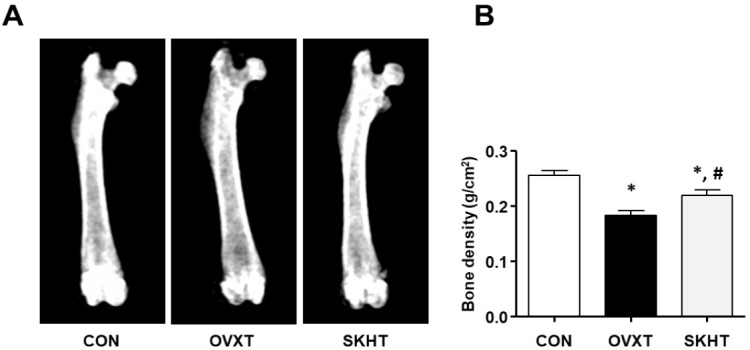
The therapeutic effect of Shinkiwhan on ovariectomy-induced rat bone loss. The bone mass was analyzed using dual energy X-ray absorptiometry (DXA). (A) Representative DXA images of the femur of the control (CON) group, ovariectomized (OVXT) group, and the group of OVX rats treated with 210 mg/kg/day Shinkiwhan (SKHT). (B) The bar graphs show the bone density. Data are expressed as mean ± standard deviation. * *P* < 0.05 versus CON group. # *P* < 0.05 versus OVX group.

The bone density of rats of each experimental group was measured using DXA. As shown in Figure 1, it was 0.2557 ± 0.0026 g/cm^2^ in CON, 0.1833 ± 0.0028 g/cm^2^ in OVXT, and 0.2196 ± 0.003 g/cm^2^ in SKHT.

### SKH regulates the glycosaminoglycan in OVX rats

**Figure 2.**
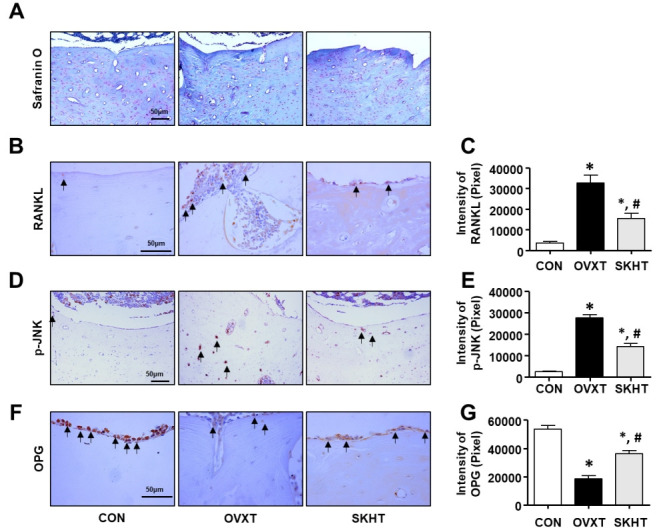
The effect of Shinkiwhan in osteoclast activity of ovariectomized rats. The rats were sacrificed and their femurs were isolated. The femurs were sectioned in 5-μm- thick slices. (A) Femurs were stained with Safranin O solution. (B, D, and F) The sections were stained with specific antibodies, such as phosphorylation of JNK (p-JNK), receptor activator of NF-kB ligand (RANKL), and osteoprotegerin (OPG). The positive signal reacts with 3,3'- diaminobenzidine and acquires a brown color. The block arrows indicate p-JNK, RANKL, and OPG positivity. (C, E, and G) The bar graphs indicate the brown intensity of the photographs in panels (B), (D), and (F), respectively. Data are expressed as mean ± standard deviation. *P < 0.05 versus CON group. # *P* < 0.05 *versus* OVX group.

The distribution of glycosaminoglycan (GAG), a component of the bone matrix, was confirmed using safranin-O-fast green staining. As shown in Figure 2A, in the OVXT group, the distribution of positive GAG in the spongy and compact bones around the bone marrow decreased. In contrast, an increase in the distribution of GAG was observed in the SKHT group.

### SKH inhibits the activity of osteoclasts in OVX rats

To investigate whether SKH can regulate the activity of osteoclasts, we performed immunohistochemistry assays with specific antibodies, such as anti-RANKL, anti-p-JNK, and anti-OPG. As shown in Figures 2B and C, the RANKL-positive response in the spongy bone of OVXT group was 33,551 ± 197/20,000,000 pixels, which was significantly increased, by 795%, compared to that of the CON group. The level of RANKL in the SKHT group was 15,280 ± 816/20,000,000 pixels, which was significantly decreased, by 53%, compared to that of the OVXT group. Next, as shown in Figures 2D and E, the p-JNK-positive response in the lamella of the compact bone of OVXT group (27,601 ± 478/20,000,000 pixel cells) increased by 1,205% compared to that of the CON group (2,497 ± 100/20,000,000 pixel cells). In the SKHT group, the positive p-JNK reactions (14,130 ± 481/20,000,000 pixel cells) showed a 48% decrease compared to that of the OVXT group. Next, as shown in Figures 2F and G, the OPG-positive response in the spongy bone of the OVXT group was 18,392 ± 674/20,000,000 pixels, which was significantly reduced, by 674%, compared to the CON group. The level of OPN in the SKHT group was 36,055 ± 713/20,000,000 pixels, which was significantly increased, by 96%, compared to the OVXT group.

### Chemical composition of SKH

**Figure 3.**
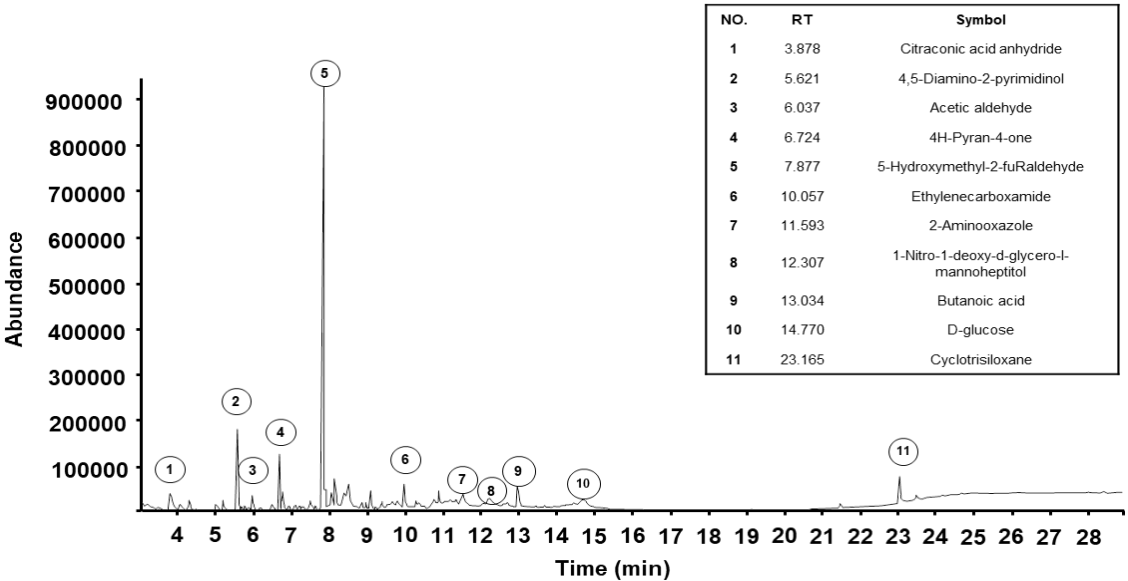
The chemical composition analysis of Shinkiwhan. RT: retention time.

Component identification was performed using gas chromatography and mass spectrometry to analyze the compounds present in SKH. A total of 11 constituents of SKH were detected, as shown in Figure 3. The components included citraconic acid anhydride (5.70%), 4,5-Diamino-2-pyrimidinol (11.51%), acetaldehyde (3.63%), 4H-Pyran-4-one (6.01%), 5-Hydroxyl-2-furaldehyde (47.68%), ethylenecarboxamide (3.72%), 2-Amino-oxazole (4.18%), 1-Nitro-1-deoxy-d-glycero-l-mannoheptitol (2.43%), butanoic acid (4.50%), D-glucose (3.63%), and cyclotrisiloxane (5.27%).

## DISCUSSION

In the present study, we evaluated whether a Korean medicine, SKH, can regulate osteoporosis in ovariectomized rats. Hence, we found that SKH had an inhibitory effect on bone mass loss in ovariectomized rats. The result was confirmed using a dual-energy X-ray absorptiometry (DXA) test. For the diagnosis of osteoporosis, a global reference standard is established by examining the BMD through X-rays of DAX. The normal value of BMD is within 1% of the standard deviation; if it is higher than 1.0% and lower than 2.5%, osteopenia is diagnosed; and if it is higher than 2.5%, osteoporosis is diagnosed [21]. In this study, the OVX model reduced the BMD by 25% compared to the normal group, whereas SKH intake showed a 15% reduction. Therefore, we suggest that SKH is effective against osteoporosis.

Bone is a physiologically active tissue that is repeatedly remodeled and regulated throughout life by osteoclasts and osteoblasts [4]. In particular, the imbalance of bone resorption and remodeling that causes osteoporosis can be caused by drugs, hereditary diseases, calcium imbalances, endocrine system abnormalities, digestive problems, and other diseases [22]. Osteoclasts play a role in bone resorption in small damaged areas of the bone [23]. In this process, RANKL expression increases through mitogen-activated protein kinase, such as phosphorylation of c-jun Nterminal kinases (JNK), and OPG performs the function of inhibiting RANKL [24]. Therefore, we suggest that SKH regulates osteoclast activity. However, these results have raised other questions about the effectiveness of SKH, including how it modulates the bone remodeling signaling pathway.

To address this question, we explored the profiles of the components of SKH using GC/MS. Our data showed that 5-hydroxyl-2-furaldehyde is a component of SKH. Tan *et al*. demonstrated that 5-hydroxyl-2-furaldehyde regulated osteogenic differentiation of bone mesenchymal stem cells [25]. Therefore, we suggest that SKH regulates bone remodeling through osteoblast differentiation.

Exercise has a good effect on maintaining overall vitality, such as heart circulation and psychological function. In particular, active people have a 50% reduction of the risk of osteophytes [26]. According to sports science reports, it is known that aerobics and weightlifting are effective in increasing the BMD of the spine or osteophytes [27]. M Y Chien *et al*. recommended an exercise protocol for bone remodeling that consists of, at least, 3 exercise sessions per week with an intensity above 70% of the maximal oxygen consumption for 30 min [28]. The molecular pathways of bone remodeling regulated by exercise have been studied and exercise-induced estrogen has been reported to inhibit bone resorption by regulating the expression of RANKL, TRPV5, and OPG [29]. However, patients with osteoporosis need to control the intensity of exercise. Osteoporosis is a disease that increases the risk of fracture due to a decreased bone density and microstructure damage in the bone tissue [30]. Therefore, we suggest that treating and preventing osteoporosis requires the intake of nutrients for bone regeneration and the use of effective health-functional substances. In addition, our further study should confirm the synergistic effect of SKH administration and exercise on bone remodeling.

## CONCLUSION

In conclusion, our findings demonstrate that SKH regulates the expression of OPG, p-JNK, and RANKL in the bones of OVX-induced osteoporosis. In addition, SKH regulates BMD in OVX rats. These results suggest that 5-hydroxyl-2-furaldehyde and the other active compounds in SKH may have protective effects against osteoporosis.
